# Governance Must Dive Into Organizations to Make a Real Difference: Comment on "Governance, Government, and the Search for New Provider Models"

**DOI:** 10.15171/ijhpm.2016.89

**Published:** 2016-07-03

**Authors:** Jean-Louis Denis, Susan Usher

**Affiliations:** ^1^Canada Research Chair in Governance and Transformation of Health Organizations and Systems, École nationale d’administration publique, Montréal, QC, Canada.; ^2^Health Innovation Forum, Montréal, QC, Canada.; ^3^Institute for Strategic Analysis and Innovation, McGill University Health Centre, Montréal, QC, Canada.; ^4^École nationale d’administration publique, Montréal, QC, Canada.

**Keywords:** Governance, Healthcare Reform, Quality Improvement

## Abstract

In their 2016 article, Saltman and Duran provide a thoughtful examination of the governance challenges involved in different care delivery models adopted in primary care and hospitals in two European countries. This commentary examines the limited potential of structural changes to achieve real reform and considers that, unless governance arrangements actually succeed in penetrating organizations, they are unlikely to improve care. It proposes three sets of levers influenced by governance that have potential to influence what happens at the point of care: harnessing the autonomy and expertise of professionals at a collective level to work towards better safety and quality; creating enabling contexts for cross-fertilization of clinical and organizational expertise, notably through teamwork; and patient and public engagement to achieve greater agreement on improvement priorities and overcome provider/manager tensions. Good governance provides guidance at a distance but also goes deep enough to influence clinical habits.

Saltman and Duran^1^ provide a timely paper based on their in-depth knowledge of health systems in Europe. They address a core dilemma in contemporary health policy, namely which governance arrangements can be adopted and implemented to improve these systems? Through case studies of hospitals in Spain and primary care in Sweden, they illustrate structural changes underway in tax-based health systems, and the challenges these raise for the governance of healthcare organizations. The “command-and-control” model described by the authors offers politicians and policy-makers few alternative mechanisms to bring about desirable changes, increasing the temptation to resort to strong levers like massive restructuring to dismantle organized and vested interests in order to overcome system inertia.^2,3^ In the last 30 years, reforms in numerous jurisdictions have produced results that often fall short of expectations,^4,5^ leaving many observers disenchanted by so-called big-bang reforms driven by politicians or “change junkies.” Health system observers find that these macro level structural changes to shake up the status quo, such as mergers, consolidation and entry of new actors have limited potential, in and of themselves, to improve care and services, and may have detrimental effects on the morale and mobilization of healthcare personnel.^6^

While it is easy to decry structural changes, finding ways to achieve real reforms is more difficult. Discussions around the governance and transformation of health systems do not take place on neutral ground; hard talk around the political economy that drives such systems is inevitable. Renewing governance and organizational forms also means rebalancing the allocation of resources in favour of broader system goals. For example, in most health systems, acute and highly specialized care still receive a much larger proportion of resources than primary care and community health interventions.

As an alternative to the hard levers of the “command and control” model, the authors see promise in governance renewal as a means of improving health systems. This entails a careful analysis and deep understanding of the key ingredients involved in the renewal of organizational forms.^7^ Put bluntly, if new governance arrangements do not manage to penetrate the meso level of organizations, they may, like restructuring, prove a poor strategy to improve care. The delivery models underlined by the authors — giving state organizations greater independence in decision-making capacity; encouraging establishment of private entities; mixing private/public market with competition^1^ — provide different governance contexts, but the models themselves need to be unpacked in order to identify and implement elements that constitute a significant and positive shift in governance for improvement. Governance must exert a greater and more appropriate influence on what happens in the clinical context and at the point of care. As Scally and Donaldson pointed out back in 1998, looking at aspirations for the “new National Health Service (NHS) in England, clinical governance requires an organization-wide transformation”^8^ (p. 61). This means paying more attention to the organization of work and the management of human resources responsible for delivering care, a lesson we learned from the socio-technical school approach to organizations more than 70 years ago.^9^

Building on the authors’ assertion that governance should reflect the practical operational realities of healthcare delivery, we propose a number of levers involving both governance and management, that can be activated to achieve improvements in care in any organizational form. Rethinking governance involves a better alignment of policies and capabilities found at the strategic and operational levels of health systems, as exemplified by work on multi-level governance.^10,11^ There are no magic recipes to ensure such alignment. However, it is clear that broad policies at the national or regional level must go beyond setting controls and targets — a point emphasized by some critics of pay for performance (P4P)^12-14^ — and actually contribute to creating a facilitative context for improvement.

Three themes come to mind in thinking about enabling contexts for improvement that appear to be influenced by governance policies. First, systems and organizations must pay serious attention to clinical leadership and engagement.^15,16^ While often discussed in reference to physicians, the focus may have to extend out to include other personnel. The autonomy and expertise of professionals needs to be harnessed at a more collective level to achieve broader system goals such as quality and safety of care.^17-19^ As Saltman found in an earlier review of reforms in European health systems, this will not happen without specific strategies and investments.^20^ The ‘black box’ of clinical governance as a process between incentive and outcome needs to be unpacked to find the instruments that can support improvement at the micro level. While organizations require the autonomy to enact these supports, no particular organizational form — public, private, not-for-profit — appears to have intrinsic advantages in achieving a better alignment of professional and organizational interests.

Secondly, while clinical leadership and engagement appear essential, this needs to be supported and nurtured by a rich work context. One of the key cultural shifts in contemporary healthcare policy and management is growing recognition of the importance of cross-fertilization between clinical and organizational assets. Bohmer has provided a plausible mapping of the attributes of high-performing clinical systems where clinical expertise and managerial know-how are blended to achieve quality improvement.^21^ Features of high-performing clinical settings include the availability of information on clinical and financial outcomes, the ability to segment the population to align care with patient and population health needs, and structures that enable learning from success and failures. More evidence is required to clearly identify robust properties of clinical contexts that are associated with significant and sustainable improvements. The message here is that organizational forms need to be animated by a clear strategy and constant commitment to improve care, and that governance has a role in enabling management to develop this enabling context. Team-based improvement efforts are a promising means of creating sustained improvement capacity, and though physician participation in this work has been difficult to achieve, it may be increased by facilitating the type of autonomous team formation based on shared purpose seen in clinical networks.^22^ A degree of flexibility would need to be incorporated into the work and compensation policies of other personnel along with physicians to allow complementary skill sets to assemble on a given challenge in an organic and regular way. Autonomy, opportunities for professional development, and mechanisms such as cross appointments to encourage new relationships across the organization^23^ have been shown to help motivate team formation, and personnel who move around an organization help bring together people with different temporal realities.^24^ Clinical and managerial leadership plays a key role in orchestrating these various levers for improvement to care processes. Governance can provide coherent incentives to support management in developing capacity-building initiatives that align organizational efforts toward improvement goals.

Finally, transforming healthcare organizations for improvement is in itself a political act. Renewing governance requires the installation of countervailing powers that counter the forces of inertia. Patient/citizen involvement and increased reliance on evidence of quality have the potential to challenge these forces. Quality indicators have multiplied in healthcare organizations, however, their ability to influence activity at the point of care has come up against barriers common in all performance management systems.^25^ Some of the more advanced organizational experiments in healthcare find that smaller quantities of highly punctual and locally relevant measurement, coupled with dialogue routines, work best to translate data into improvement.^26^ Public and patient involvement on governance bodies and in front-line improvement efforts also shows potential for countering inertia and increasing responsiveness. Use of patient experience at decision-making levels emphasizes the impact of quality gaps and increases motivation for improvement.^23,27^ Researchers looking at patient and public involvement find it leads to greater agreement about improvement priorities^28^ and may overcome some of the professional hierarchies and provider/manager tensions that have been known to hinder improvement work.^29^ Putting new actors into play and increasing the contestability of health system activities and habits are management strategies that require strong governance support. Governing healthcare is about creating well-designed organizational forms that provide supportive contexts for fostering capabilities. Results are far from guaranteed. A lack of continuity in policies can jeopardize local improvement efforts. We commend Saltman and Duran for opening this debate on the right governance model for health systems. It provides a very promising starting point for the work of unpacking the mechanisms through which governance can create facilitative contexts for improvement. This is a subtle and demanding task: learning will take time and no dramatic improvements can be expected by simply reshaping the boundaries or proprietary status of healthcare organizations. Good governance implies simultaneously steering activities at a distance, and going deep and granular enough to foster good clinical habits.

## Ethical issues

Not applicable.

## Competing interests

Authors declare that they have no competing interests.

## Authors’ contributions

Both authors were equally involved in developing the concepts and finalizing the text of this article.

## Authors’ affiliations

^1^Canada Research Chair in Governance and Transformation of Health Organizations and Systems, École nationale d’administration publique, Montréal, QC, Canada. ^2^Health Innovation Forum, Montréal, QC, Canada. ^3^Institute for Strategic Analysis and Innovation, McGill University Health Centre, Montréal, QC, Canada. ^4^École nationale d’administration publique, Montréal, QC, Canada.
